# Inhibition of *Plasmodium falciparum* Hsp70-Hop partnership by 2-phenylthynesulfonamide

**DOI:** 10.3389/fmolb.2022.947203

**Published:** 2022-09-13

**Authors:** Tshifhiwa Muthelo, Vhahangwele Mulaudzi, Munei Netshishivhe, Tendamudzimu Harmfree Dongola, Michelle Kok, Stanley Makumire, Marianne de Villiers, Adélle Burger, Tawanda Zininga, Addmore Shonhai

**Affiliations:** ^1^ Department of Biochemistry & Microbiology, University of Venda, Thohoyandou, South Africa; ^2^ Department of Biochemistry, Stellenbosch University, Matieland, South Africa; ^3^ Structural Biology Research Unit, Department of Integrative Biomedical Sciences, University of Cape Town, Cape Town, South Africa

**Keywords:** *Plasmodium falciparum*, molecular chaperone, PfHsp70-1, PfHop, inhibitor, PES, pifithrin μ

## Abstract

*Plasmodium falciparum* Hsp70-1 (PfHsp70-1; PF3D7_0818900) and PfHsp90 (PF3D7_0708400) are essential cytosol localized chaperones of the malaria parasite. The two chaperones form a functional complex via the adaptor protein, Hsp90-Hsp70 organizing protein (PfHop [PF3D7_1434300]), which modulates the interaction of PfHsp70-1 and PfHsp90 through its tetracopeptide repeat (TPR) domains in a nucleotide-dependent fashion. On the other hand, PfHsp70-1 and PfHsp90 possess C-terminal EEVD and MEEVD motifs, respectively, which are crucial for their interaction with PfHop. By coordinating the cooperation of these two chaperones, PfHop plays an important role in the survival of the malaria parasite. 2-Phenylthynesulfonamide **(**PES) is a known anti-cancer agent whose mode of action is to inhibit Hsp70 function. In the current study, we explored the antiplasmodial activity of PES and investigated its capability to target the functions of PfHsp70-1 and its co-chaperone, PfHop. PES exhibited modest antiplasmodial activity (IC_50_ of 38.7 ± 0.7 µM). Furthermore, using surface plasmon resonance (SPR) analysis, we demonstrated that PES was capable of binding recombinant forms of both PfHsp70-1 and PfHop. Using limited proteolysis and intrinsic fluorescence-based analysis, we showed that PES induces conformational changes in PfHsp70-1 and PfHop. In addition, we demonstrated that PES inhibits the chaperone function of PfHsp70-1. Consequently, PES abrogated the association of the two proteins *in vitro*. Our study findings contribute to the growing efforts to expand the arsenal of potential antimalarial compounds in the wake of growing parasite resistance against currently used drugs.

## 1 Introduction

It is estimated that malaria accounted for 627,000 deaths in 2020 (World Health Organisation, 2021). The latest data show that malaria deaths increased by over 200,000 deaths possibly on account of a lack of commitment to managing the disease in the wake of the COVID-19 pandemic. In addition, there are increasing reports of resistance against currently used antimalarial drugs. There is therefore urgent need to expand the arsenal of antimalarial compounds. The malaria parasite traverses between a cold-blooded mosquito vector and the warm-blooded human host and thus undergoes multiple physiological changes during its complex life cycle. As part of its survival strategy, the parasite relies on its heat shock protein (Hsp) machinery to adapt to constant physiological changes and stress associated with its development (Pallavi et al., 2010; Shonhai, 2010). In addition, Hsps are implicated in antimalarial drug resistance (Witkowski et al., 2010).

**FIGURE 1:**
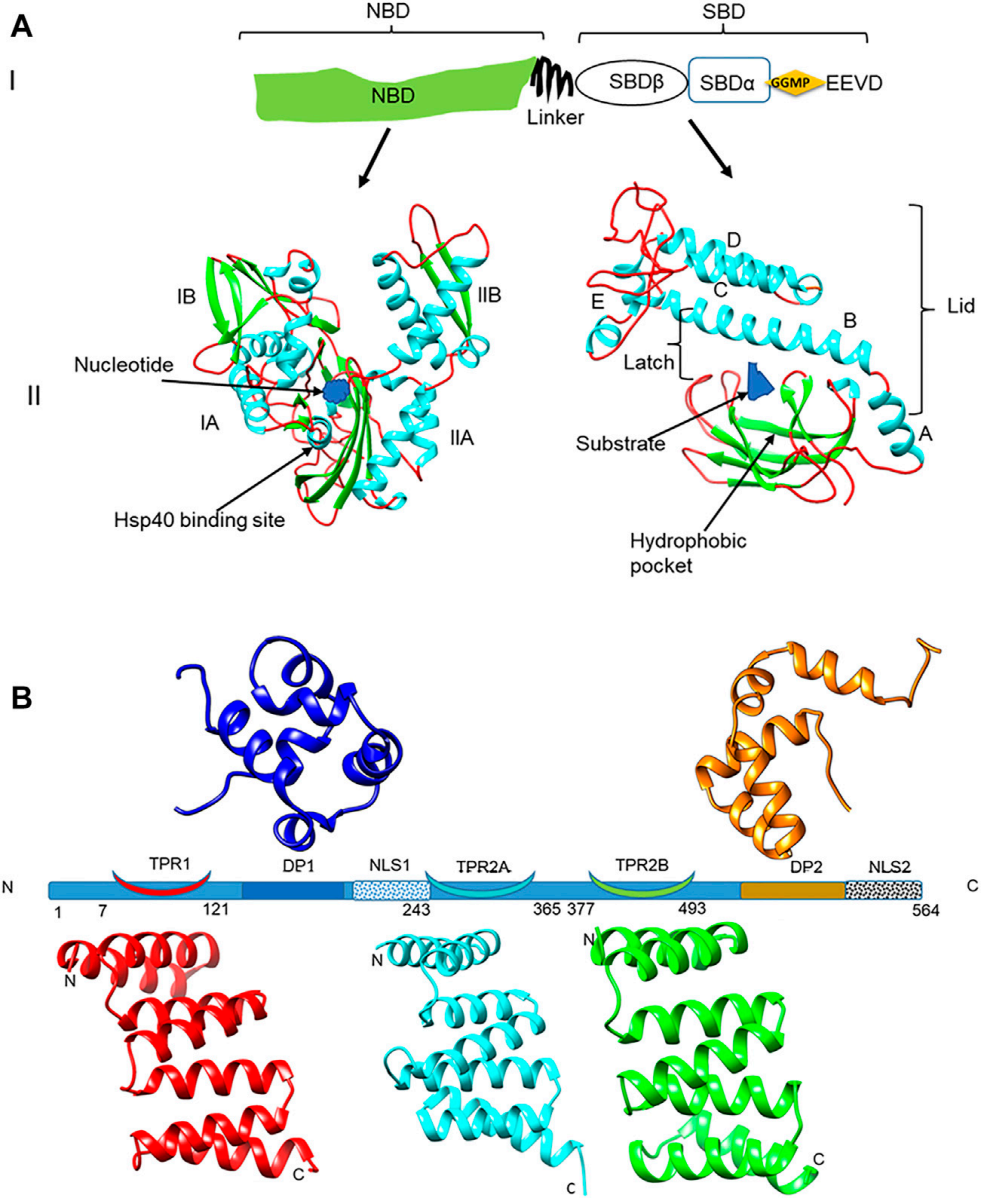
Domain representation of PfHsp70-1 and PfHop **(A)** (panel I): representation of the linear structural organization of PfHsp70-1 showing the NBD, a highly charged linker, and the-SBD. The C-terminal EEVD motif is illustrated. Panel II: Three-dimensional model of PfHsp70-1 domains. The N-terminal NBD which is subdivided into lobes IA, IB, IIA, and IIB, respectively, is shown. Also illustrated are the bound nucleotide (blue) and the Hsp40 co-chaperone binding site, respectively. The C-terminal SBD of the protein is shown on the right hand side. The hydrophobic pocket located in the SBD and a bound peptide substrate are also shown. The alpha-helical lid which is made up of helices A, B, C, D, and E is also depicted as adapted from Shonhai (2007). **(B)** Depiction of the structural organization of PfHop showing the relative positions of the three tetracopeptide repeat (TPR) regions, the two dipeptide domains (DP), and the two nuclear localization signals (NLS) of the protein. Ribbon representations of the three-dimensional models of TPR1, TPR2A, TPR2B, DP1, and DP2 domains are also shown. The models were generated using PHYRE^2^ (http://www.sbg.bio.ic.ac.uk/phyre2; Kelley et al., 2015) and rendered using CHIMERA version 1.15rc (Pettersen et al., 2004).

Hsp70 is regarded as the most abundant molecular chaperone found in all major organelles (Saibil, 2013). Hsp70 is structurally divided into two major domains; a 45 kDa N-terminal nucleotide binding domain (NBD) that exhibits ATPase activity and a 25 kDa substrate binding domain (SBD) that binds the target client protein (Mashaghi et al., 2016) (Figure 1A). *P. falciparum* Hsp70-1 (PfHsp70-1; PF3D7_0818900) is an essential molecular chaperone that is resident within the parasite cytosol (Kappes et al., 1993; Shonhai, 2021). PfHsp90 (PF3D7_0708400) co-localizes with PfHsp70-1 and the two proteins form a chaperone complex (Gitau et al., 2012) that is thought to coordinate the folding of select proteins that are important for the development of the parasite. In addition, the interaction of PfHsp70-1 and PfHsp90 is modulated by PfHop (PF3D7_1434300; Gitau et al., 2012; Zininga et al., 2015) (Figure 1B), which serves as a module that facilitates substrate exchange between the two chaperones.

Both PfHsp70-1 and PfHsp90 are essential proteins (Banumathy et al., 2003; Lu et al., 2020), suggesting a possible essential role for PfHop. Due to their critical role in the development of the parasite, both PfHsp70-1 and PfHsp90 are deemed prospective antimalarial drug targets (reviewed in Zininga and Shonhai, 2019; Daniyan, 2021). However, targeting *P. falciparum* Hsps is a challenge, as these proteins are generally conserved (Pesce et al., 2010; Chakafana et al., 2019). Despite this, there is growing evidence that Hsp70s of parasitic origin, and in particular PfHsp70-1, exhibit distinct structure-function features that could make them amenable to selective targeting (Chakafana et al., 2019; Anas et al., 2020; Lebepe et al., 2020; Makumire et al., 2021). Promisingly, some antimalarial compounds demonstrating selective inhibition of parasite Hsp70 with minimum effects on the function of human Hsp70 have been described (Cockburn et al., 2014; reviewed in Zininga and Shonhai, 2019). One of the attractive features of targeting PfHsp70-1 is that its inhibition abrogates both its independent chaperone function as well as its association with co-chaperones (Zininga et al., 2017a, b). This, and growing evidence pointing to the role of Hsps in antimalarial drug resistance (Akide-Ndunge et al., 2009; Corey et al., 2016), justifies efforts to target them in antimalarial drug development efforts as they hold promise especially as co-targets in combination therapies.

2-Phenylthynesulfonamide (PES), also known as pifithrin µ, is a member of the benzene family. PES is an anticancer agent that is known to target human Hsp70 (Leu et al., 2009; Jiang and Xiao, 2020). However, the mechanism of action of PES on the Hsp70 function is not fully understood. It has been suggested that it binds to the NBD of Hsp70 (Zhou et al., 2017), and this contradicts earlier findings proposing that it binds to the C-terminal SBD (Leu et al., 2009). The antimalarial activity of PES and its effect on the function of PfHsp70-1 remains to be established. In the current study, we investigated the effect of PES on asexual blood-stage *P. falciparum* parasites and we established that it exhibits modest antiplasmodial activity. Furthermore, we observed that PES binds directly to both PfHsp70-1 and PfHop, inducing conformational changes in both proteins. Consequently, PES abrogated the chaperone activity of PfHsp70-1, and further inhibited its association with the co-chaperone, PfHop. We discuss the implications of our findings with respect to both antimalarial drug discovery efforts and the possible mechanism of action of PES.

## 2 Experimental procedures

### 2.1 Materials

Unless otherwise stated, the materials used in this study were purchased from Sigma-Aldrich (Darmstadt, Germany), Merck Millipore (Darmstadt, Germany), and Melfords (Ipswich, United Kingdom). The following antibodies were used in the study: α-PfHsp70-1 (Shonhai et al., 2008), α-PfHop (Gitau et al., 2012), and α-His (Thermofisher, MA, United States).

### 2.2 *In silico* docking of PES onto PfHsp70-1 and PfHop

To predict the possible binding, as well as identify possible binding sites of PES on PfHop and PfHsp70-1, AutoDock Vina (Trott and Olson, 2010, http://vina.scripps.edu) was used. The structures used for the docking were obtained from homology modeling using AlphaFold’s ColabFold (Mirdita et al., 2021). As templates, the crystal structures of the NBD (6S02 and 6RZQ, Day et al., 2019; 7P31, 7OOH, 7OOG, 7OOE,7NQZ, 7NQU, 7NQS and 7NQR, Mohamad et al., 2021a–g) and SBD (6ZHI, Schmidt and Vakonakis, 2020) of PfHsp70-x bound to various ligands were used to predict the structure of PfHsp70-1. These were specified as custom templates. The structure of PfHop was predicted without any custom templates specified. Both the protein and ligand were prepared for docking using AutoDockTools4 (Morris et al., 2009). Default parameters were used with minor modifications. The active site was placed in a grid box with x, y, and z grid points set at 100 while the grid point spacing was 0.375 Å. The energy range was set at 4 and the exhaustiveness set at 100. Docking was initiated using command prompt. Upon completion of the docking, an output file with 9 binding modes was generated together with a log file scoring the modes in terms of binding affinity (in kcal/mol). The output files (in pdbqt format) were viewed in Pymol™ 2.4.1 (The PyMOL Molecular Graphics System, Schrodinger, LLC, NY, United States) and converted to PDB files. The receptor-ligand interactions were then performed in Discovery Studio Visualizer Version 20.1.0.19295; (BIOVIA, Sandiego, CA, United States) and images were generated using LigPlot+, version 2.2.4 (Wallace et al., 1995).

### 2.3 Expression and purification of recombinant proteins

Production and purification of recombinant forms of both PfHsp70-1 and PfHop protein was conducted as previously described (Makumire et al., 2020, 2021). Briefly, the plasmid constructs for pQE30-PfHsp70-1 and pQE30-PfHop were transformed into *E. coli* XL1 Blue cells. An additional construct, pQE30-PfHsp70-1_NBD_ encoding for the NBD of PfHsp70-1 was similarly used to express the truncated version of this chaperone (Zininga et al., 2016). The production of recombinant protein was induced by isopropylthiogalactoside (IPTG) and the protein was purified using sepharose nickel affinity chromatography (Makumire et al., 2020, 2021). The expression and purification of recombinant proteins were analyzed by SDS-PAGE and confirmed by Western blot analysis using α-PfHsp70-1 (Shonhai et al., 2008), α-PfHop (Gitau et al., 2012), and α-His antibodies, respectively.

### 2.4 Investigation of the effect of PES on the conformation of PfHsp70-1 and PfHop using limited proteolysis

Limited proteolysis has previously been used to validate the nucleotide-dependent conformational alterations of PfHsp70-1 (Zininga et al., 2016). In the current study, we employed the same approach to explore the effects of PES on the fragmentation of the recombinant forms of PfHsp70-1 and PfHop. Fragmentation of PfHsp70-1 or PfHop in the presence of nucleotides served as controls. Briefly, recombinant PfHsp70-1 (4 µM) or PfHop (4 µM) was digested with 0.25 ng/ml of proteinase K at 37 C in the absence and presence of 25 µM ADP/ATP or 20 µM PES for 30 min. Proteolytic digestion of either PfHsp70-1 or PfHop was analyzed using SDS-PAGE analysis followed by silver staining using GE Healthcare PlusOne™ Silver Staining Kit (WI, United States).

### 2.5 Intrinsic fluorescence-based analysis of the effect of PES on the tertiary structures of PfHsp70-1 and PfHop

The effect of PES on the tertiary structural conformation of recombinant PfHsp70-1 and PfHop proteins relative to the nucleotide-dependent conformations were assessed by monitoring intrinsic fluorescence as previously described (Zininga et al., 2016; Lebepe et al., 2020). Briefly, recombinant PfHsp70-1 (4 µM) or PfHop (4 µM) was incubated in the absence or presence of 25 µM ATP/ADP. The assay was repeated in the presence of 20 µM PES. Fluorescence emission spectra were monitored at 300–400 nm after initial excitation at 295 nm. The spectra data collected from seven spectral scans were averaged and processed taking into account the baseline (effect of buffer in the absence of protein).

### 2.6 Analysis of the effect of PES on the chaperone activity of PfHsp70-1

The capability of PfHsp70-1 to prevent thermal aggregation of malate dehydrogenase (MDH) from porcine heart was previously demonstrated (Shonhai et al., 2008). In the current study, we investigated the effect of PES on the holdase chaperone activity of PfHsp70-1. The assay was initiated by adding 1,25 µM MDH and 0,75 µM PfHsp70-1 to the assay buffer (50 mM Tris-HCl, 100 mM NaCl; pH 7.4) and heated to 51°C. Aggregation of MDH was monitored as a function of light scattering at 360 nm over 30 min at 51°C in a SpectraMax M3 (Molecular Devices, United States) microplate spectrometer. The assay was repeated at varying final concentrations (5, 15, 25, 50, 100 nM) of PES. The chaperone function of PfHsp70-1 under various experimental conditions was compared to the activity of the chaperone recorded in the absence of nucleotide. Statistical analysis was conducted using a student t-test and a *p* **<** 0.05 represented functionally significant variation.

### 2.7 Determination of equilibrium binding kinetics of PES to either PfHop or PfHsp70-1

The steady-state equilibrium binding kinetics for the inhibitors on either PfHsp70-1 or PfHop were investigated using BioNavis Navi 420A ILVES multi-parametric surface plasmon resonance (MP-SPR) system (BioNavis, Finland) following a previously described method (Lebepe et al., 2020; Chakafana et al., 2021). Briefly, filter-sterilized de-gassed PBS (4.3 mM Na_2_HPO_4_, 1.4 mM KH_2_PO_4_, 137 mM NaCl, 3 mM KCl, 0.005% (v/v) Tween 20, and 20 mM EDTA; pH 7.4) was used as running buffer for the assay. The ligand (0.1 μg/ml of PfHsp70-1/PfHop) was immobilized onto a carboxymethyl dextran (CMD 3-D) gold sensor chip through amine coupling. PES was injected (flow rate of 50 μl/min) as analyte at varying final concentrations (0, 1.25, 2.5, 5, 10, 20 nM). Similarly, as controls nucleotides (5 µM ATP/ADP) and a known *P. falciparum* Hsp70 inhibitor, (−)-Epigallocatechin-3-gallate (EGCG; Zininga et al., 2017b) were similarly injected at varying final concentrations (0, 1.25, 2.5, 5, 10, 20 nM) over the chip surface. Steady-state equilibrium was achieved after allowing the interaction to occur for 8 min at 25°C, followed by dissociation for 4 min at 25°C, respectively. The data generated were analyzed using Data Viewer (BioNavis, Finland) after subtraction of baseline (signals generated on the chip surface without protein immobilized and buffer without inhibitor). The resultant sensorgrams were analyzed to determine the equilibrium binding affinities using Trace Drawer software version 1.8 (Ridgeview Instruments; Sweden). A student t-test *p* **<** 0.05 represented statistically significant differences in affinity recorded relative to the activity of the protein reported in the absence of nucleotide.

### 2.8 Analysis of the effect of PES on the association of PfHop and PfHsp70-1

#### 2.8.1 Surface plasmon resonance assay

To determine the effect of inhibitors on the direct interaction of PfHsp70-1 with PfHop (Gitau et al., 2012; Zininga et al.,2015) we conducted SPR analysis using the BioNavis Navi 420A ILVES MP-SPR system (BioNavis, Finland). PfHsp70-1 was immobilized as ligand and varying concentrations (0, 125, 250, 500, 1,000, and 2000 nM) of PfHop as analyte were injected on the chip surface at a flow rate of 20 μl/min. To monitor the effects of the inhibitor, the analyte was suspended in PBS supplemented with 25 µM PES and injected similarly. We previously established that EGCG abrogates PfHop-PfHsp70-1 interaction (Zininga et al., 2017b). As such, as control, the assay was repeated in the presence of 25 µM of EGCG in place of the PES. Association was allowed to occur for 5 min, followed by dissociation for 10 min at 25°C. Data analysis was conducted taking baseline correction into account through double referencing [subtracting the signals recorded using both buffer blank (PBS supplemented with 25 µM of inhibitor without the analyte protein) and the chip blank (channel with BSA as ligand protein immobilized)]. The generated sensorgrams were then analyzed as described in Section 2.7 above. The affinity values obtained under the various experimental conditions were compared to that observed for the assay conducted in the absence of nucleotide.

#### 2.8.2 Enzyme-linked immunosorbent assay

An enzyme-linked immunosorbent assay (ELISA) was used to validate the effect of PES on the direct association of PfHsp70-1 and PfHop following a previously described protocol (Mabate et al., 2018). Briefly, ligand (5 μg/ml of PfHsp70-1) suspended in 5 mM sodium bicarbonate (NaHCO_3_) at pH 9.5 was noncovalently immobilized onto a 96 well plate overnight at 4 C. BSA (5 μg/ml) was used as a ligand for the negative control. The wells were washed with 150 µl TBST which contains (Tris-buffered saline- Tween (TBST; 20 mM Tris HCl, pH 7.5, 500 mM NaCl supplemented with 0.1% Tween 20) prior to blocking with 5% fat-free milk in TBST and incubated at 25 C for 1 h. The wells were washed using TBST for a total of 10 min. Varying concentrations (0–1,000 nM) of analyte PfHop were added to the wells and incubated for 2 h at 25 °C. To remove the unbound analyte, the plate was washed with 150 µl TBST three times before the addition of rabbit-raised α-PfHop antibody (1: 4,000) followed by incubation at 25°C for 1 h. Subsequently, the wells were washed with 150 µl TBST three times before the addition of 100 µl secondary HRP conjugated goat-raised α-rabbit antibody (1: 4,000) and incubated for 45 min at 25 °C. The substrate, 3,3’5,5’ –tetramethylbenzidine (TMB) (Bio Scientific, United States) was added into the wells and incubated for 2 min at 25 C. Color development was monitored by recording absorbance readings at 370 nm at 5 min time intervals for 30 min using a SpectraMax M3 Microplate reader (Molecular Devices, United States). To determine the effect of the inhibitor, the assay was conducted in the presence of 25 µM) of PES. As controls, the assay was repeated in the presence of 25 µM of ATP/ADP and EGCG (Zininga et al., 2015; Zininga et al., 2017b). Furthermore, the assay was repeated interchanging the ligand and analyte to validate data reproducibility. The generated data were analyzed taking into account the background signal generated by BSA which served as the control protein. The absorbance values obtained at the highest concentration of each analyte were averaged to represent maximum (100%) binding. Titration curves were then generated using GraphPad Prism 9.3.1 (GraphPad Software, United States). The relative binding affinities of PES under each experimental setting were normalized relative to the affinity estimated for assay conducted in the absence of nucleotide at the highest concentration of PfHop. Statistical analysis was conducted to establish significant differences at *p* < 0.05 using a student t-test.

### 2.9 Parasite growth inhibition studies

Asexual *P. falciparum* strain 3D7 parasites were cultured at 2–3% parasitemia in RBC (4% haematocrit) in RPMI complete media supplemented with 25 mM HEPES, 11 mM glucose, 200 μM hypoxanthine (dissolved in 500 mM NaOH), 24 μg/ml gentamycin and 0.6% m/v Albumax II serum. Growth inhibition assays were set up on synchronized ring-stage parasites using a 1% parasitemia and a 1% haematocrit suspension. The culture was exposed to increasing concentrations of PES (1.95–500 μM) and incubated at 37 °C in a continuous gas environment (93% N_2_, 3% O_2_, and 4% CO_2_) for 96 h. Chloroquine was used as the positive drug control at 1 μM. Parasite growth without any drug present, which allowed the parasites to proliferate unrestricted, was used as negative drug control. SYBR™ Gold DNA stain (Invitrogen, ThermoFisher Scientific Inc., Germany) was used to measure the proliferation of the parasites compared to the controls as previously described (Smilkstein et al., 2004). Fluorescence was measured using a TECAN Spark^®^ multimode microplate reader (Tecan Trading AG, Switzerland) and the data were analyzed using SigmaPlot, version 12 (Systat Software Inc., IL, United States). IC_50_ were generated based on dose-response curves obtained as technical triplicates for one biological test (n = 1 for chloroquine) and three biological repeats (n = 3 for PES).

## 3 Results

### 3.1 PES is predicted to directly bind to both PfHsp70-1 and PfHop

**FIGURE 2:**
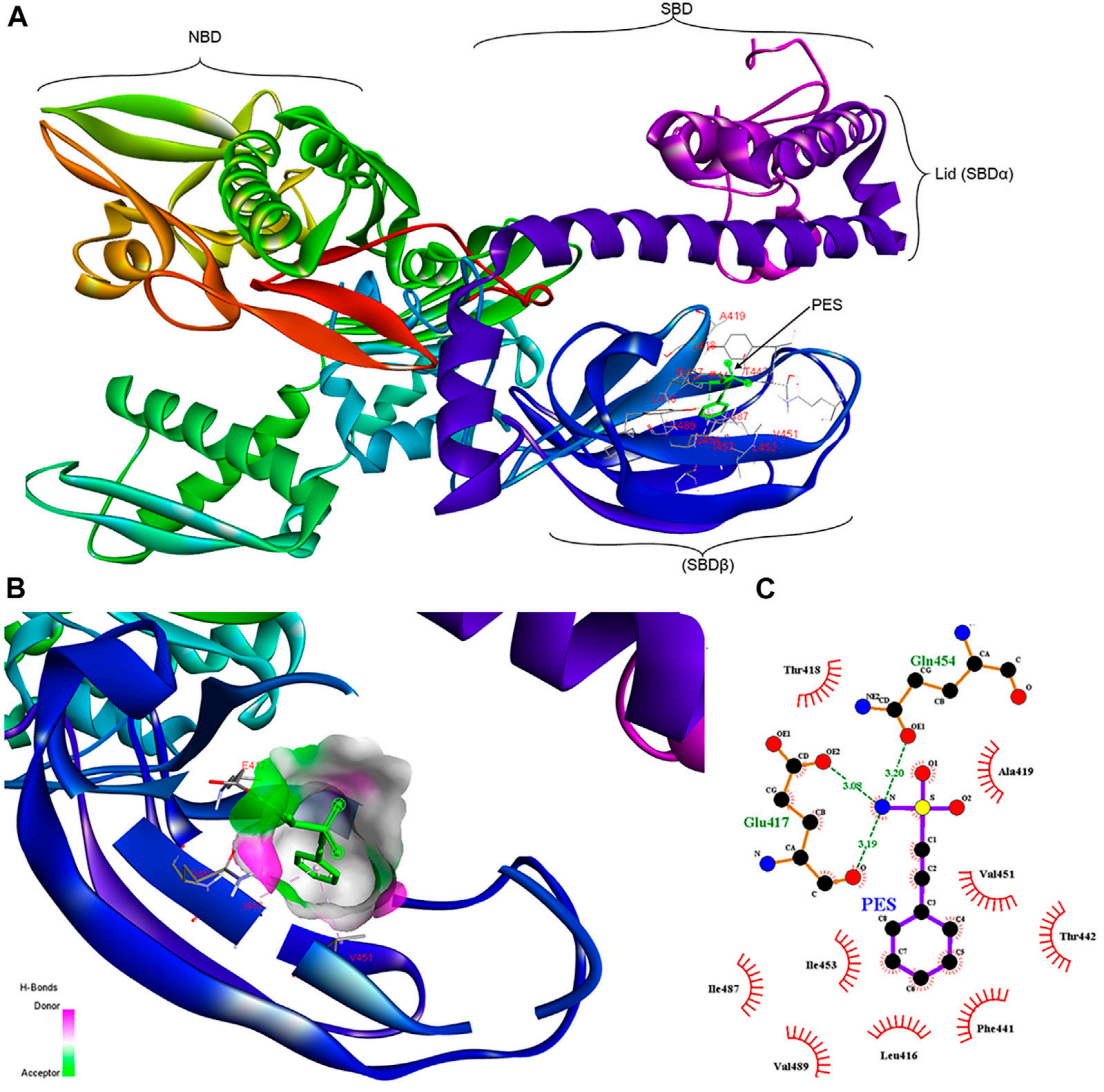
Schematic representing the interaction of PES with PfHsp70-1.**(A)** representation of a three-dimensional model with a ribbon structure of full-length PfHsp70-1 bound to PES through the SBDβ. The different domains of PfHsp70-1 are also shown. **(B)** Surface and ribbon views of the docked structures, showing the residues involved in the interaction, and **(C)** a 2D diagram showing receptor-ligand interface. PES makes H bond contacts with Glu417, Gln454 of PfHsp70-1. In addition, PES exhibits pi-alkyl contacts with Val451 and Ile453 of PfHsp70-1.

**FIGURE 3:**
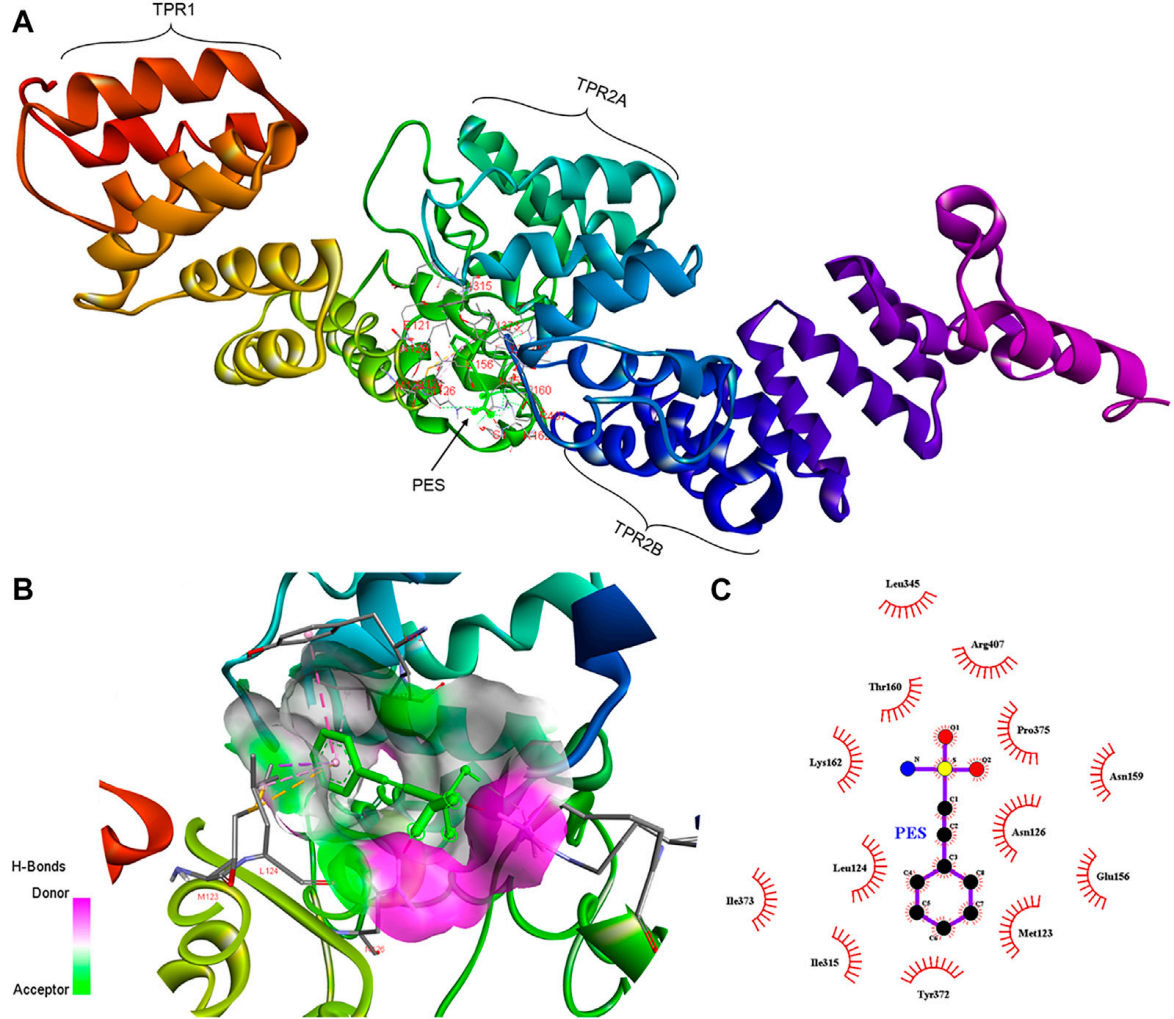
Schematic representing the interaction of PES with PfHop. **(A)** A docked three-dimensional model with a ribbon structure of full-length PfHop bound to PES. The domains of the protein are also shown. PES binds to the TPR1, DP1, and TPR2B regions of the protein. **(B)** Surface and ribbon views of the docked structures, showing the residues involved in the interaction. **(C)** A 2D illustration of the receptor-ligand interface. PES appears to hydrogen bond with residues Arg407 on PfHop. Thus, PES forms pi-pi T-shaped interactions with Tyr372, pi-alkyl with Leu124 and Ile373, pi sigma, and pi-sulfur with Met123 of PfHop.

Molecular docking studies were conducted to assess the propensity of PES to bind to PfHsp70-1 and its co-chaperone, PfHop. PES was docked onto PfHsp70-1 and PfHop and the affinity was determined in each case (Figures 2, 3). Both PfHsp70-1 and PfHop bound to PES at notable predicted scores of -6.9 kcal/mol and 7.3 kcal/mol, respectively. The predicted binding of PfHop by PES came as a surprise since this compound is traditionally known to target Hsp70. Based on the docking model, PES is positioned within a binding pocket defined by residues Leu416, Glu417, Thr418, Ala419, Phe441, Thr442, Val451, Ile453, Gln454, Ile487, and Val489 located within the SBD of PfHsp70-1 (Figure 2). Our findings are thus in agreement with a previous study that proposed that PES binds to the SBD of human Hsp70 (Leu et al., 2009). It is thought that the substrate binding cavity of PfHsp70-1 is made up of residues Ala419, Tyr444, and Val451 (Shonhai et al., 2007; Shonhai et al., 2008). It is interesting to note that residues, Ala419 and Val451 that form part of the substrate binding cavity of PfHsp70-1 are predicted to make direct contact with PES. It is plausible that PES binding by PfHsp70-1 may abrogate substrate binding.

Furthermore, PES is predicted to interact with two residues, Met123 and Leu 124, located within the interface between the TPR1 and the dipeptide domain 1 (DP1) of PfHop (Figure 3). In addition, PES is thought to make direct contact with both Tyr372 and Ile373 which are located within the region joining TPR2A and TPR2B of PfHop. Residue Arg407, located within the TPR2B of PfHop seems to also make direct contact with PES (Figure 3). The predicted binding energies of PES for the two proteins are proportional to the estimated number of H-bond and the net number of interactions observed; 5 for PfHsp70-1 and 6 for PfHop, respectively (Figure 3; Table 1). The marginally higher binding affinity of PES for PfHop could be attributed to the distinct contacts it makes with this co-chaperone relative to PfHsp70-1 (Table 1).

**TABLE 1:** Interaction of PES with *P. falciparum* Hsp70-1/PfHop as predicted *in silico*.

Protein	Compound	Binding energy (Kcal/mol)	Hydrogen bonding and residues involved	Hydrophobic interactions and residues involved
PfHop	PES	−7.3	Arg407	Met123, Leu124, Tyr 372, Ile373
PfHsp70-1		−6.9	Glu417, Gln454	Val451, Ile453

### 3.3 Experimental evidence for the direct binding of PES onto PfHsp70-1 and PfHop

**FIGURE 4:**
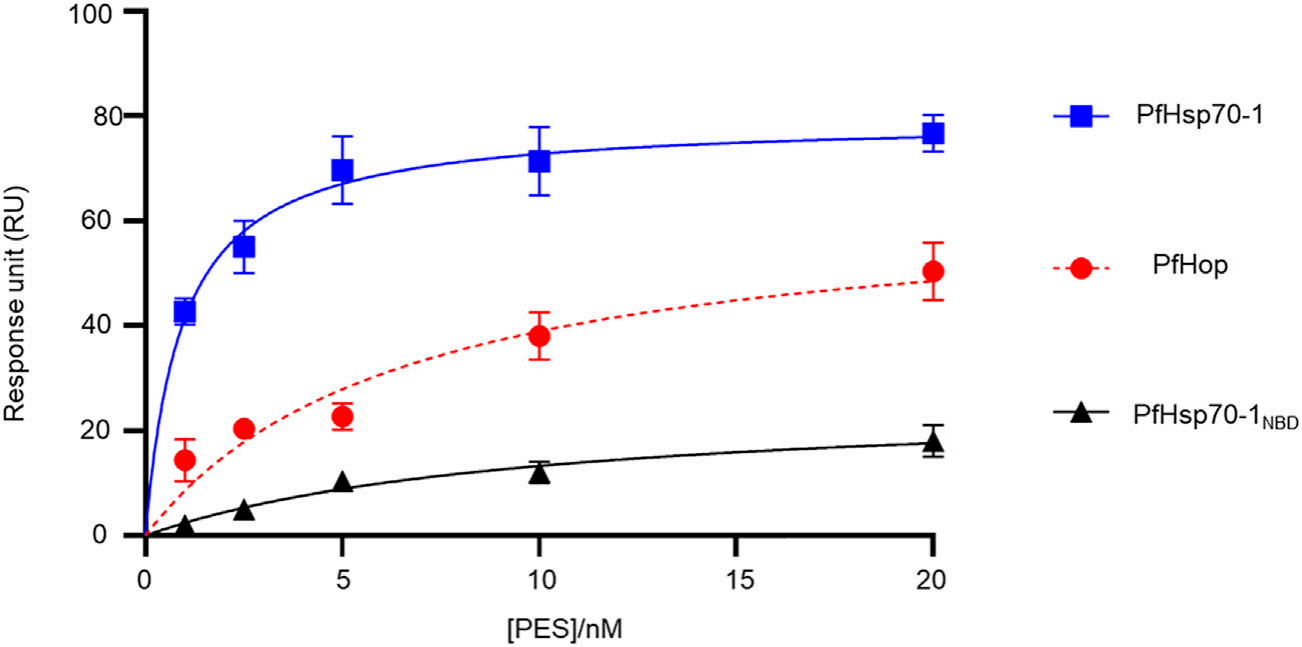
PES directly binds to both PfHsp70-1 and PfHop. As a ligand, each of PfHsp70-1 or PfHsp70-1_NBD_ or PfHop was immobilized at a concentration of 0.1 μg/ml. The assay was conducted in the presence of variable levels of PES. The interaction between ligand and analyte was determined at equilibrium. Each curve is the average determined for three independent experiments, each performed in triplicate. The error bars are indicated as standard error about the mean.

We further experimentally investigated the direct interaction of PES with both PfHsp70-1 and PfHop. We employed SPR analysis to explore the association of PES with either of the two proteins. Recombinant forms of either PfHsp70-1 or PfHop were used as analytes while PES served as ligand. First, it was important to validate the interaction of PfHsp70-1 with ATP (Table 2) as the latter is known to bind to PfHsp70-1 (Zininga et al., 2016). In addition, while human Hop binding to ATP has been reported (Yamamoto et al., 2014), the direct interaction of PfHop with nucleotides has not been established. As expected, PfHsp70-1 and its NBD both bound to ATP (Table 2). It is interesting to note that PfHop bound to ATP within affinity of the same order of magnitude as PfHsp70-1 (Table 2). This is the first report demonstrating that PfHop, like human Hop, is capable of binding ATP. *In silico* prediction suggests that PES binds primarily to the TPR2A subdomain of PfHop (Figure 3). However, this requires experimental validation. As expected, PfHsp70-1 bound to PES within the lower micromolar range (Table 2). On the other hand, the isolated NBD of PfHsp70-1 exhibited much less affinity (about two orders of magnitude lower) for PES, further confirming that PES binding is largely driven by residues located in the SBD (Figure 4). PfHop bound to PES with notable affinity, although its affinity for the ligand was one order of magnitude lower than PfHsp70-1.

**TABLE 2:** Equilibrium binding affinities of PES for PfHsp70-1/PfHsp70-1_NBD_ and PfHop.

Ligand	Analyte	KD (µM)	χ^2^
PfHsp70-1	PES	0.0692 ± 0.02	1.70
	ATP	0.537 ± 0.07	1.73
PfHop	PES	0.707 ± 0.07	7.31
	ATP	0.861 ± 0.01	6.90
PfHsp70-1_NBD_	PES	7.39 ± 0.09	9.14
	ATP	0.0223 ± 0.03	1.01

*K*_*D*_, equilibrium constant, X^2^ values indicate the goodness of fit for SPR, sensorgrams. Each parameter is the mean for three independent experiments, each performed in triplicate and standard errors of the mean are indicated.

Our SPR data (Figure 4; Table 2) is at variance with docking studies that suggested that PES exhibits a higher affinity for PfHop than PfHsp70-1 (Table 1). In addition, findings from the docking studies suggest that PES binds to Hsp70 via both the NBD and the SBD. While the minor discordances between *in silico* and experimental data were expected, both the *in silico* and experimental data strongly suggested that PfHop binds to PES. While the recognition of PES by the SBD of Hsp70 has been established (Leu et al., 2009, 2011; Kaiser et al., 2011), evidence for its possible interaction with the N-terminal NBD of Hsp70 has been reported (Zhou et al., 2017). Thus, our findings suggest that PfHsp70-1 is capable of binding PES via both domains reconciling the previously contrasting reports.

### 3.2 PES induces conformational changes in PfHsp70-1 and PfHop

**FIGURE 5:**
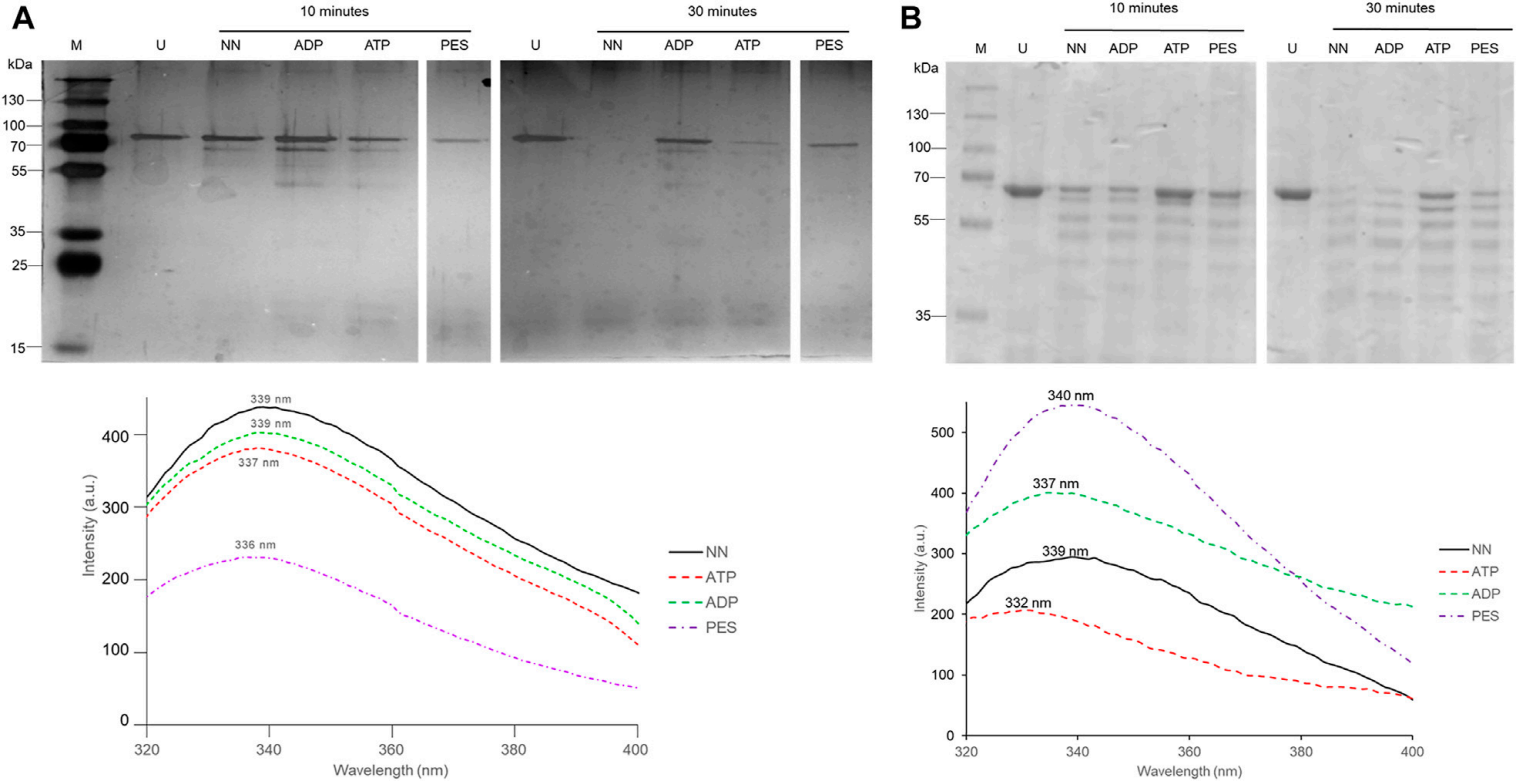
PES induces conformational changes in PfHsp70-1. Recombinant PfHsp70-1 **(A)** protein was subjected to limited proteolysis (top panel) and intrinsic fluorescence analysis (lower panel) in the presence of ATP/ADP and PES. Similarly, recombinant PfHop protein **(B)** was subjected to limited proteolysis (top panel) and intrinsic fluorescence analysis (lower panel) in the presence of ATP/ADP and PES. Fluorescence emission spectra were averaged for seven spectral scans monitored at 310–400 nm after an initial excitation of 295 nm.

The capability of PES to induce conformational changes on PfHsp70-1 and PfHop was analyzed using limited proteolysis and intrinsic fluorescence analysis. The effect of ATP/ADP on the fragmentation of PfHsp70-1 or PfHop served as a control. The data generated through limited proteolysis and intrinsic fluorescence suggest that ATP and ADP each uniquely regulate the conformations of either PfHsp70-1 (Figure 5A) or PfHop (Figure 5B). While it is well known that PfHsp70-1 is uniquely regulated by ATP and ADP (Zininga et al., 2016), this study constitutes the first report suggesting that the conformation of PfHop is regulated by nucleotides. We further explored the structural perturbations of both proteins in the presence of PES. Both PfHsp70-1 and PfHop digested in the presence of PES generated unique fragmentation patterns compared to protein digested in *apo* state (Figure 5A,B). This suggests that PES binds to PfHsp70-1 and PfHop to induce conformational changes. Similarly, intrinsic fluorescence data mirrored the same findings as we noted a blue shift (emission peak of 336 nm) registered by PfHsp70-1 in the presence of PES relative to the emission peak of 339 nm registered by protein in *apo* state (Figure 5A bottom panel). On the other hand, PfHop registered a marginal red shift (emission peak of 340 nm) in the presence of PES relative to the emission peak of 339 nm registered by the protein in *apo* state (Figure 5B bottom panel).

### 3.4 PES inhibits the holdase chaperone activity of PfHsp70-1

**FIGURE 6:**
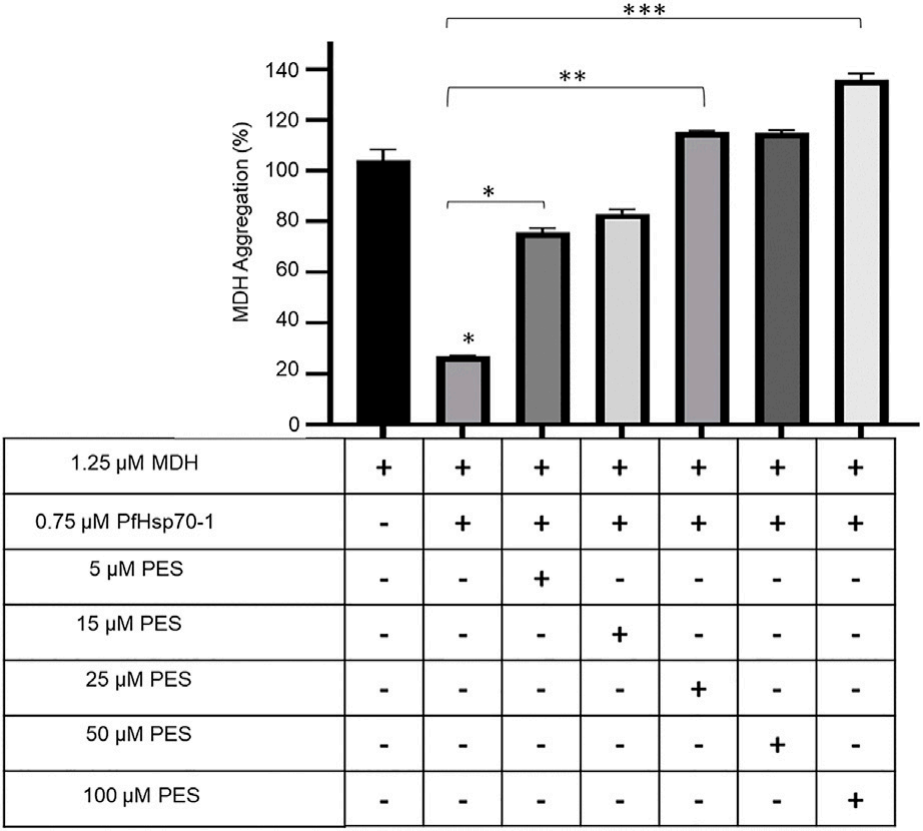
PES inhibits the holdase chaperone activity of PfHsp70-1. The effect of PES on the heat-induced aggregation suppression activity of PfHsp70-1 was monitored by exposing aggregation-prone protein, MDH, to heat stress at 51 °C in the presence of PfHsp70-1 at equimolar levels and varying levels of PES. The heat-induced aggregation of MDH was monitored at 360 nm. Error bars indicate the mean generated from three assays conducted using independent PfHsp70-1 protein purifications. Statistical significance of differences was determined using one-way ANOVA and post-hoc test (*p < 0,05; **p < 0,01; ***p < 0.001).

PfHsp70-1 is known to suppress the heat-induced aggregation of model substrate proteins such as MDH, thereby exhibiting holdase chaperone activity (Shonhai et al., 2008; Makumire et al., 2021). We explored the effect of PES on the capability of PfHsp70-1 to suppress the heat-induced aggregation of MDH *in vitro*. The rationale of this assay is that inhibition of PfHsp70-1 would result in MDH aggregating in the presence of the chaperone. Our findings demonstrated that PES inhibited the chaperone activity of PfHsp70-1 in a concentration-dependent fashion (Figure 6).

### 3.5 PES inhibits the direct association of PfHsp70-1 and PfHop

**FIGURE 7:**
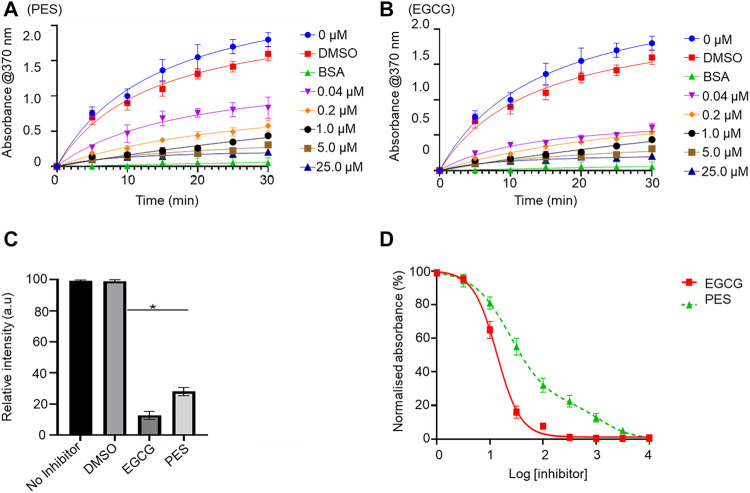
PES suppresses PfHop-PfHsp70-1 association. PfHsp70-1 was immobilized onto the ELISA plate and PfHop (analyte) was suspended along with varying concentrations of PES. DMSO and BSA were used as controls. Representative binding curves obtained for the association of PfHsp70-1 and PfHop in the presence of **(A)** PES and EGCG **(B)** are shown. Bar graphs showing the comparative effects of the inhibitors on the interaction of PfHsp70-1 and PfHop are provided **(C)**. The resultant dose-response curves for both PES and EGCG are shown **(D)**. The error bars represent the standard deviations obtained from three independent assays conducted independently. Student t-test statistical significance of differences of inhibitor compared to the vehicle control, DMSO, are indicated by asterisks positioned above the bar graphs (*p* < 0.001*).

The capability of PES to inhibit the direct association of PfHsp70-1 with PfHop was investigated using ELISA and SPR analyses as previously reported (Mabate et al., 2018). First, using ELISA, we established that PES inhibited the association of the two proteins in a concentration dependent fashion irrespective of which protein was used as a ligand (Supplementary Figure S1). BSA was used as a negative control protein as it does not interact with either PfHsp70-1 or PfHop (Supplementary Figure S1). The assay was repeated in the presence of 25 µM of either ATP or ADP (Supplementary Figure S1) or 25 µM inhibitor (Figure 7; Table 3). The presence of ADP promoted the association, while ATP abrogated the association (Supplementary Figure S1). This observation was in line with our previous findings (Zininga et al., 2015; Zininga et al., 2017a; Zininga et al., 2017b), thus validating the assay. PES abrogated the interaction of PfHsp70-1 and PfHop in the same way as ATP (Figure 7A). The assay was further validated using EGCG (Figure 7B), a known inhibitor of PfHop-PfHsp70-1 interaction (Zininga et al., 2017b). This suggests that PES has an inhibitory effect on the association of PfHsp70-1 with PfHop as is the case for ATP or EGCG (Figure 7C). In addition, the ELISA-based data suggested that compared to EGCG, PES is marginally less effective at inhibiting PfHop-PfHsp70-1 interaction (Figure 7D).

**TABLE 3:** PES inhibits the direct association of PfHsp70-1 and PfHop.

Ligand	Analyte	Name of the inhibitor	IC_50_ (nM)
PfHsp70-1	PfHop	EGCG	13.90 ± 1.2
		PES	32.43 ± 2.1
PfHop	PfHsp70-1	EGCG	14.57 ± 1.8
		PES	38.13 ± 3.6

Interaction of PfHsp70-1 and PfHop was investigated using ELISA, with analyte and ligand alternating. IC_50_ values for the interaction of the proteins in the presence of PES and EGCG (control) are shown.

**FIGURE 8:**
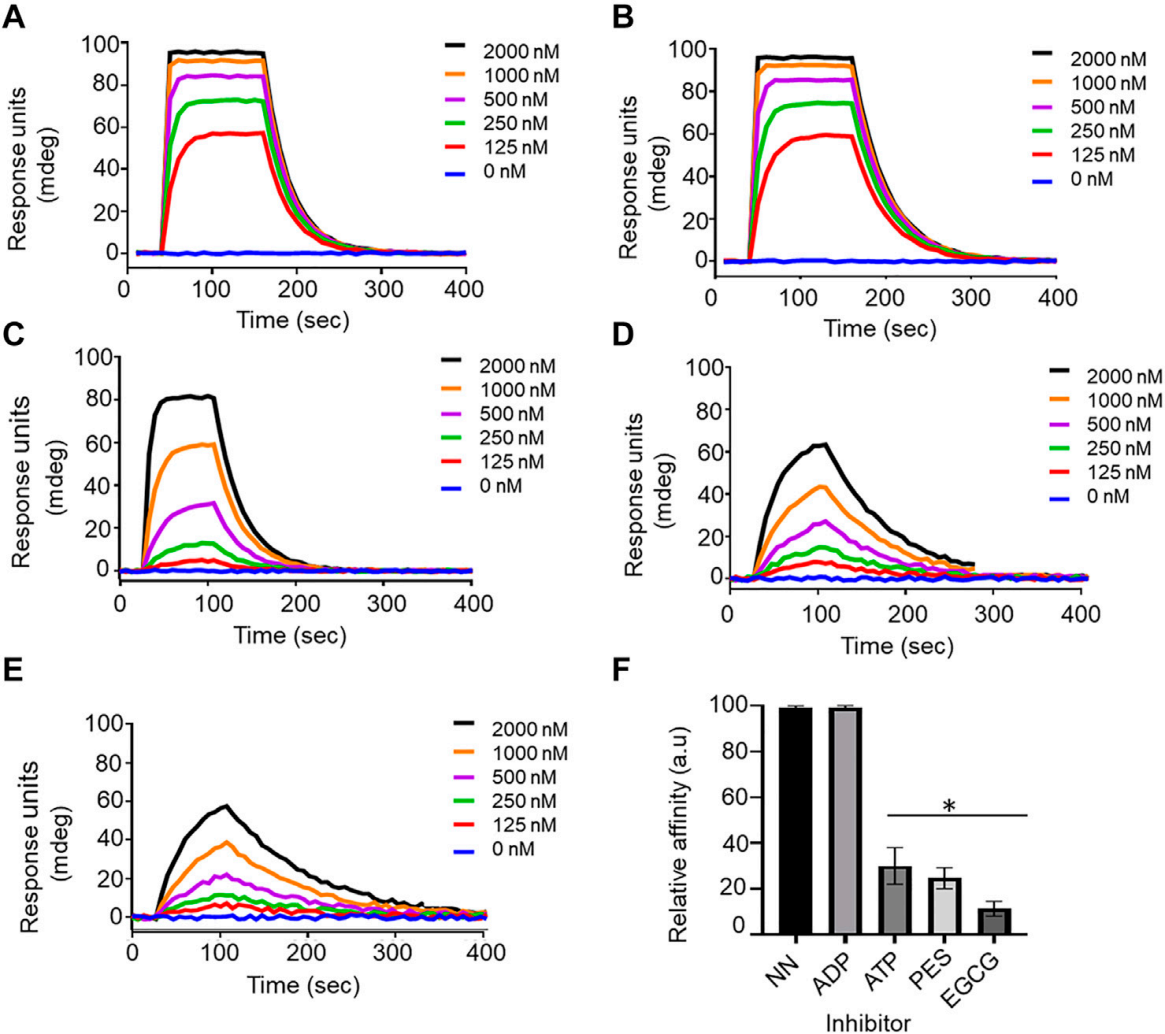
Confirmation of the inhibitory function of PES on PfHop-PfHsp70-1 association. Representative sensorgrams generated from data obtained in absence of nucleotide **(A)**, presence of ADP **(B)**, ATP **(C)**, and PES **(D)** and EGCG **(E)** are shown. The relative affinities of the association of PfHop with PfHsp70-1 in the absence (NN) of both nucleotides and inhibitors compared to interaction in the presence of nucleotides and inhibitors are shown in **(F)**. Standard errors represent three independent assays conducted in triplicates using independent protein batches. A student t-test was used for statistical validation and statistically significant differences relative to data obtained in the absence of nucleotide state (NN) are indicated by asterisks above the bar graphs (*p* < 0.001*).

We further validated the effect of PES on the PfHop-PfHsp70-1 association using SPR analysis. PfHsp70-1 was immobilized as ligand and varying concentrations of PfHop were injected as analyte. The assay was conducted in the absence or presence of 25 µM ATP/ADP, or PES (Figure 8). First, we confirmed that under the SPR-based experimental conditions PfHop and PfHsp70-1 associate in a nucleotide-dependent fashion (ADP promotes their association while ATP inhibits the association; Zininga et al., 2015; Figure 8). Our SPR-based data confirmed that PES significantly inhibited the interaction of PfHop with PfHsp70-1 (Figure 8). However, as observed using the ELISA assay, PES was slightly less effective at this than EGCG (Figures 7, 8).

To further validate the inhibition of PfHsp70-1 and PfHop interaction, ligand and analyte were switched, and the assay was repeated (Table 4). As previously shown, a decrease in response was observed in the interaction of PfHsp70-1 with PfHop in the presence of PES. The binding affinities for PfHsp70-1 with PfHop in the presence of PES were within the same order of magnitude after swapping ligand and analyte (Table 4). Taken together, both our SPR and ELISA data suggest that PES abrogates the PfHop-PfHsp70-1 association.

**TABLE 4:** SPR based kinetics showing the effect of PES on the interaction of PfHsp70-1 with PfHop.

Ligand	Analyte	Nucleotide/Inhibitor	*ka* (1/Ms)	*kd* (1/s)	*K*_*D*_ (nM)	χ^2^
PfHsp70-1	PfHop	NN	7.67 (±0.07) e^5^	3.25 (±0.05) e-^3^	4.24 ± 0.4	0.91
		ADP	7.81 (±0.01) e^5^	3.08 (±0.08) e^−3^	3.08 ± 0.8	0.75
		ATP	2.59 (±0.09) e^5^	2.07 (±0.07) e^−2^	79.8 ± 8.0^*^	2.12
		EGCG	6.31 (±0.01) e^3^	1.30 (±0.3) e^−1^	20,700 ± 700^*^	0.51
		PES	1.25 (±0.05) e^5^	2.66 (±0.06) e^−2^	214 ± 40^*^	0.13
PfHop	PfHsp70-1	NN	7.60 (±0.6) e^6^	3.41 (±0.01) e^−2^	4.49 ± 0.9	0.19
		ADP	8.07 (±0.07) e^6^	2.50 (±0.5) e^−2^	3.10 ± 0.1	0.10
		ATP	2.66 (±0.06) e^5^	2.15 (±0.05) e^−2^	80.9 ± 9.1^*^	1.67
		EGCG	6.71 (±0.01) e^3^	9.14 (±0.04) e^−3^	1,360 ± 60^*^	0.12
		PES	3.50 (±0.5) e^4^	8.11 (±0.01) e^−3^	232 ± 24^*^	0.28

*ka*, association rate constant; *kd*, dissociation rate constant; *K*_*D*_, equilibrium constant; X^2^ values indicate the goodness of fit for SPR, sensograms based on the bivalent fit model. Three independent analyses were conducted during the SPR, assay alternating ligand and analyte each time. Standard errors are shown in brackets. Statistical analysis was done using a student t-test (*p* **<** 0.05)* represents statistically significant differences in affinities recorded in the absence of nucleotide (NN).

### 3.6 The antiplasmodial activity of PES

**FIGURE 9:**
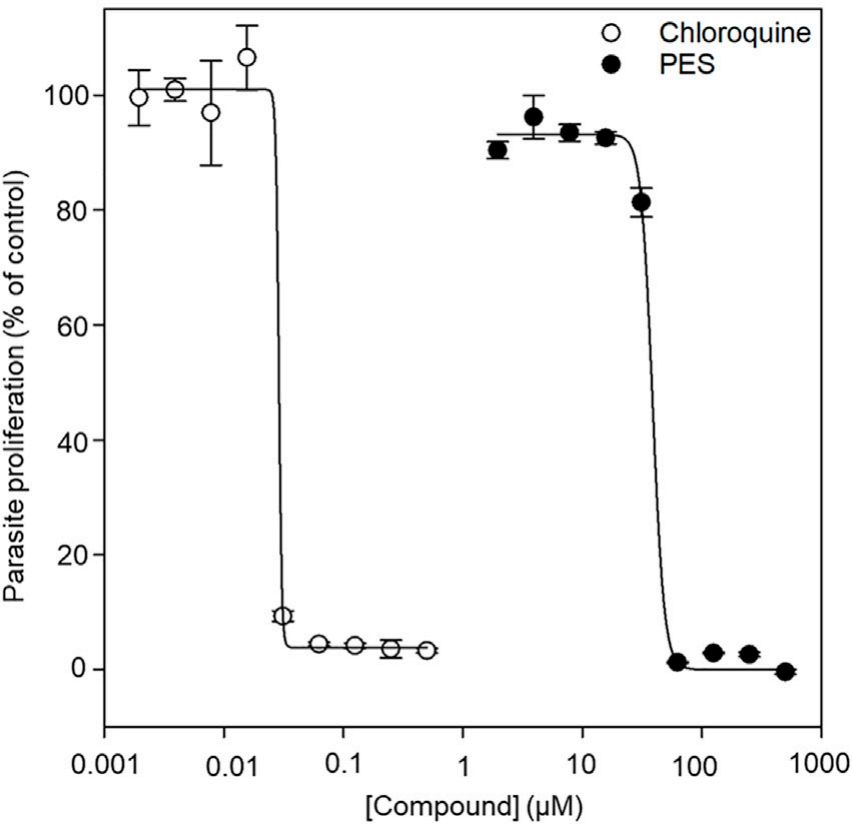
The normalized dose-response curve for the *in vitro* susceptibility of *P. falciparum* to PES. The *in vitro* antiplasmodial activity of PES was investigated using a SYBR™ Gold DNA stain to measure the proliferation of the parasites as compared to the controls. Fluorescence was measured using a TECAN Spark^®^ multimode microplate reader and the data analysis was done using SigmaPlot, version 12 (Systat Software Inc., Chicago, IL, United States). IC_50_ determinations were performed as technical triplicates for three biological repeats with standard error of the mean shown.

We further explored the capability of PES to inhibit *P. falciparum* proliferation at the asexual blood stage. The antiplasmodial activity of PES was compared to that of chloroquine which is a known antimalarial (Zininga et al., 2017b). In the presence of PES, *P. falciparum* 3D7 parasite growth was inhibited in a concentration dependent manner with an IC_50_ value of 38.7 ± 0.7 µM compared to chloroquine with an IC_50_ value of 29.1 ± 1.3 nM (Figure 9). The IC_50_ of chloroquine compares well with published potencies (21 ± 1.7 nM, Smilkstein et al., 2004). On the other hand, PES exhibited mild antiplasmodial activity.

## 4 Discussion

*P. falciparum* causes the most severe form of malaria in humans. The effectiveness of antimalarial drugs has been reduced due to emerging drug-resistant parasite strains. As such, there is a need to explore novel drug targets for the treatment of malaria. PfHsp70-1 and PfHsp90 are two prominent and essential molecular chaperones of the parasite. The two chaperones functionally cooperate via PfHop mediation (Gitau et al., 2012; Zininga et al., 2015). PES has been reported to possess antitumour activity (Leu et al., 2009; Kaiser et al., 2011; Zhou et al., 2017) and offers promise toward repurposing as a potential agent to fight the growing threat of multidrug-resistant pathogens. It has been proposed that PES binds to the SBD of Hsp70 (reviewed in Moradhi-Marjaneh et al., 2019). This study is the first to show that PES binds to PfHsp70-1 to inhibit its chaperone function. Furthermore, PES inhibits the interaction of PfHsp70-1 with the co-chaperone, PfHop. Whereas the interaction of PES with Hsp70 has been established, this study for the first time demonstrated that PES binds to both PfHsp70-1 and PfHop.

**FIGURE 10:**
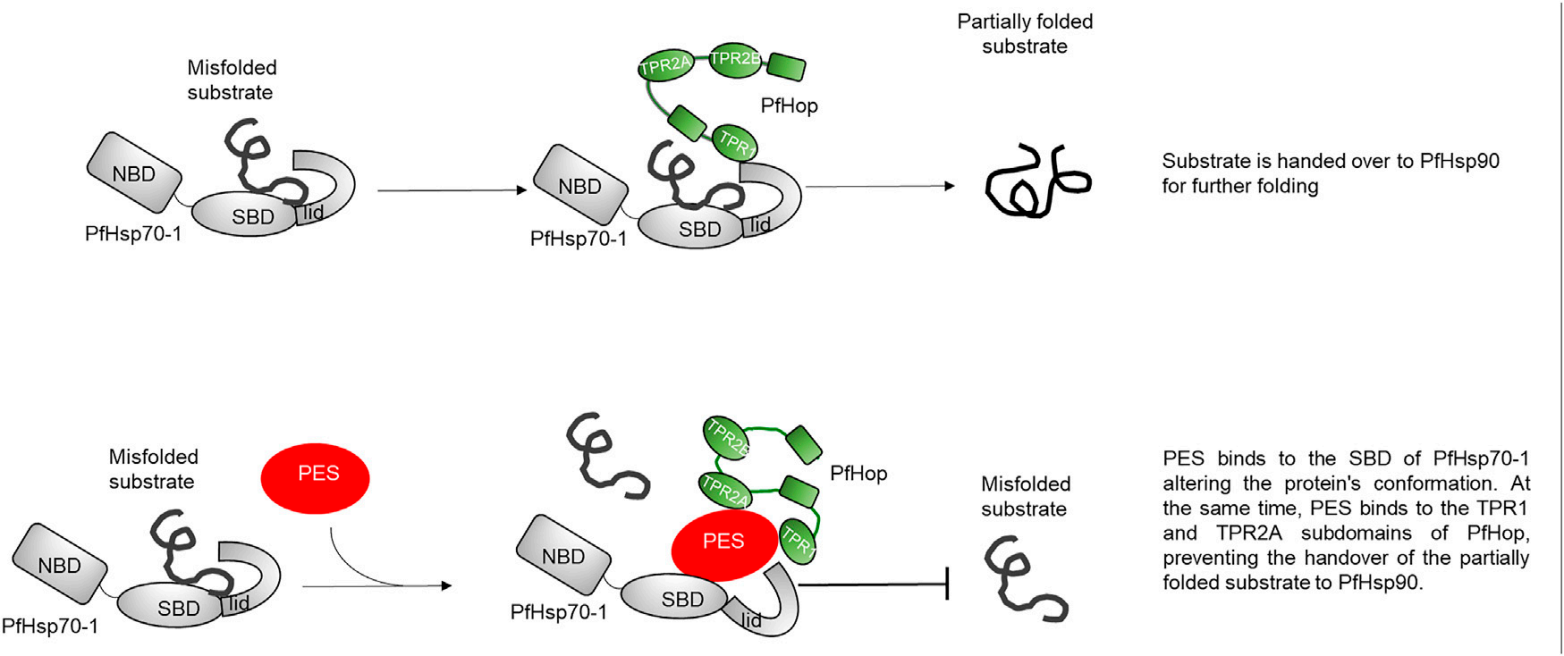
Proposed model of PES binding PfHsp70-1. The schematic represents a model for the mechanism of action of PES. The model suggests that PES binds to both PfHsp70-1 and PfHop thereby perturbing the conformations of the two proteins. Consequently, this abrogate the chaperone function of PfHsp70-1 and their interaction. The inhibition of the interaction of PfHsp70-1 and PfHop adversely impacts on the functional partnership between PfHsp70-1 and PfHsp90.

First, we conducted *in silico* docking studies and observed that PES is predicted to bind to both proteins. It has been reported that PES binds to the SBD of human Hsp70 (Leu et al., 2009). We further demonstrated that the binding of PES onto PfHsp70-1 induces a conformational switch that is distinct compared to that of the protein in the *apo* state (Figure 5). Similarly, a conformational change was observed for PfHop in the presence of PES as compared to the protein in the *apo* state (Figure 5). In addition, the *in silico* data suggested that PES binds the co-chaperone, PfHop via contact residues located within the TPR1:DP1 and, TPR2A:TPR2B interfaces of the protein. We previously resolved the structure of PfHop using SAXS analysis. Our previous study demonstrated that the TPR domains of PfHop assemble like “beads on a string’’ (Makumire et al., 2020). This arrangement allows Hsp70 and Hsp90 to slide through the concave-shaped TPR domains to facilitate interaction. Thus, PES may abrogate the association of PfHop with PfHsp70-1 by perturbing the conformation of both PfHop and PfHsp70-1. Having established that PES forces PfHsp70-1 to assume a conformational switch, we further enquired if this would impact the chaperone activity of PfHsp70-1. PES abrogated the holdase chaperone activity of PfHsp70-1 (Figure 6). Apart from inducing a conformational switch, PES may also physically block substrate binding since our *in silico* data suggested that this compound interacts with residues located within the C-terminal substrate binding domain, including those constituting the hydrophobic pocket (Val451) and the arch (Ala419 and Tyr444) which are all crucial for substrate binding (Figure 10; Shonhai et al., 2008). Furthermore, our data demonstrated that PES binds to both proteins and hence we speculate that the dual binding role of PES may account for its capability to abrogate the interaction of the two proteins (Figure 10).

We further established that PES inhibited the growth of *P. falciparum* parasites maintained at the blood stage registering a modest IC_50_ of 38.7 µM (Figure 9). The inhibition of the PfHsp70-1-PfHop pathway may account for the observed antiplasmodial activity. It has been suggested that about 24% of malarial proteins possess prion-like asparagine repeat-rich segments, thus, the parasite proteome may have propensity to aggregate under heat stress (Muralidharan et al., 2012; Pallarès et al., 2018). This makes the role of the molecular chaperone machinery of the parasite crucial for survival in the host.

Altogether, our findings demonstrate that PES binds both PfHsp70-1 and PfHop to disrupt their association as well as abrogate the chaperone function of PfHsp70-1. In addition, that PES is predicted to bind to TPR and DP1 subdomains of PfHop while possibly making contacts with several residues located in the SBC of PfHsp70-1 makes it an important scaffold for designing versatile chemical inhibitors targeting this pathway. Since PES has been shown to inhibit Hsp70 in cancer cells, its selectivity for parasites versus the human homolog may be in question. Hence, further work needs to be conducted to establish its utility in the fight against both malaria and cancer. Future efforts must focus on the crystallization of both PfHsp70-1 and PfHop in complex with PES. A thermal shift assay of the protein-ligand complexes would also serve as an appropriate precursor step to establish the prospects of successful crystallization of the complexes.

## Data Availability

The raw data supporting the conclusions of this article will be made available by the authors, without undue reservation.
