# Supplementary material for: Inhibition of Plasmodium falciparum Hsp70-Hop partnership by 2-phenylthynesulfonamide
Source: Front Mol Biosci. 2022 Sep 13;9:947203. doi: 10.3389/fmolb.2022.947203 (PMC9513230; doi:10.3389/fmolb.2022.947203)
Supplement: Supplementary file 1 [file DataSheet1.docx]

Supplementary Material

## Supplementary Figures


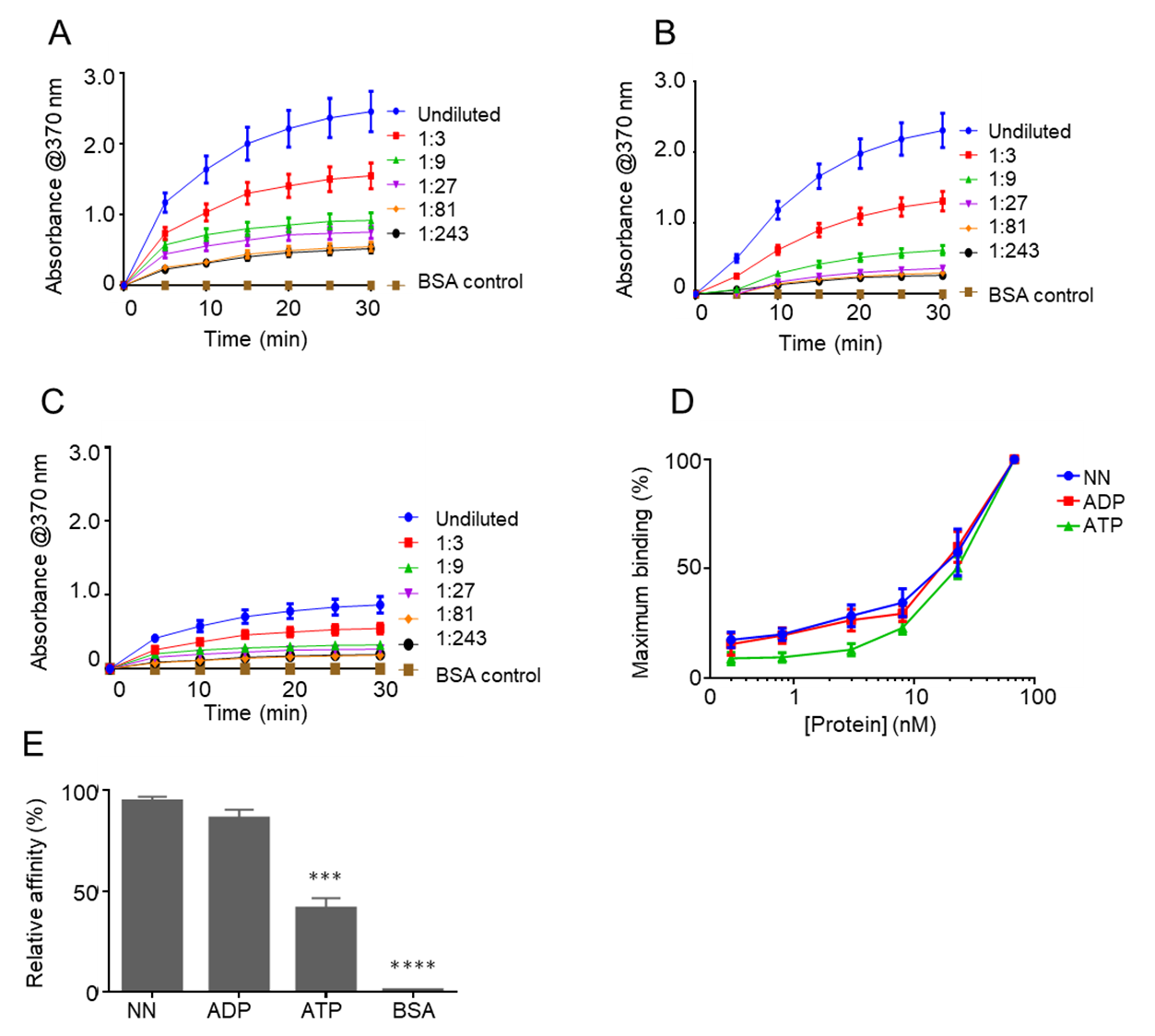


**Supplementary Figure S1. ELISA for the interaction of PfHsp70-1 with PfHop**

PfHsp70-1 was immobilized onto the ELISA plate as ligand. PfHop was passed over the immobilized chaperone. BSA was used as control. Representative binding curves obtained for the association of PfHsp70-1 and PfHop in the absence of nucleotide (**A**), and presence of either ADP (**B**) or ATP (**C**) are shown. The dose-response curves of the IC_50_ values of nucleotides is shown (**D**). Bar graphs showing the comparative effects of the nucleotides on the interaction of PfHsp70-1 and PfHop are shown (**E**). The error bars represent the standard deviations obtained from three independent assays conducted. ANOVA statistical significance of differences of inhibitor compared to the control are indicated by asterisks positioned above the bar graphs (p<0.05***; p<0.001****).
